# Metabolic tumor volume and the survival of patients with Non-Hodgkin lymphoma treated with chimeric antigen receptor T cell therapy: a meta-analysis

**DOI:** 10.3389/fimmu.2024.1433012

**Published:** 2024-08-29

**Authors:** Lin Liu, Feng Jin, Hua Fan

**Affiliations:** ^1^ Department of Hematology, The Forth Affiliated Hospital of China Medical University, Shenyang, China; ^2^ Department of Breast Surgery, The First Affiliated Hospital of China Medical University, Shenyang, China

**Keywords:** Non-Hodgkin lymphoma, chimeric antigen receptor T, metabolic tumor volume, positron emission tomography, survival

## Abstract

**Background:**

Chimeric antigen receptor T cell (CAR-T) is a promising treatment for aggressive Non-Hodgkin lymphoma (NHL). The aim of the meta-analysis was to determine the association between metabolic tumor volumes (MTV) derived on positron emission tomography before CAR-T infusion and the survival of patients with NHL.

**Methods:**

Relevant observational studies pertaining to the purpose of the meta-analysis were obtained through a search of PubMed, Web of Science, and Embase from inception of the databases to April 1, 2024. The data was combined using a random-effects model that accounted for the potential influence of between-study heterogeneity.

**Results:**

Fifteen observational studies were included. Pooled results showed that compared to those with a lower MTV, the NHL patients with a higher MTV before CAR-T infusion were associated with a poor progression-free survival (hazard ratio [HR]: 1.73, 95% confidence interval [CI]: 1.48 to 2.02, p < 0.001; I^2 =^ 20%) and overall survival (HR: 2.11, 95% CI: 1.54 to 2.89, p < 0.001; I^2 =^ 58%). Subgroup analysis showed that the association between MTV and survival of NHL patients after CAR-T was not significantly impacted by study design, methods for determination of MTV cutoff, or analytic models (univariate or multivariate, p for each subgroup all < 0.05). Subgroup analysis suggested a stronger association between MTV and poor survival outcomes in patients with median of lines of previous treatment of 2 or 3 as compared to those of 4 (p for subgroup difference < 0.05). Further meta-regression analyses suggested that the association between MTV and survival was not significantly affected by sample size, age, proportion of men, cutoff value of MTV, follow-up duration, or study quality scores (p all > 0.05).

**Conclusion:**

A high MTV at baseline is associated with a poor survival of NHL patients after CAR-T.

**Systematic Review Registration:**

https://inplasy.com/, identifier INPLASY (INPLASY202450069).

## Introduction

Non-Hodgkin lymphoma (NHL) represents a heterogeneous group of lymphoid malignancies characterized by the proliferation of abnormal lymphocytes (1, 2). It ranks among the most prevalent hematologic malignancies globally, with its incidence steadily rising over the past few decades (3, 4). NHL encompasses various subtypes, each with distinct clinical features, prognoses, and treatment responses (5). While advancements in therapy have improved outcomes for many patients, the management of aggressive NHL subtypes remains challenging. Among the emerging treatment modalities, chimeric antigen receptor T cell (CAR-T) therapy has garnered significant attention for its remarkable efficacy in treating relapsed or refractory NHL (6, 7).

CAR-T therapy involves genetically modifying patients’ T cells to express chimeric antigen receptors targeting specific antigens, such as CD19, expressed on the surface of lymphoma cells (8, 9). Upon infusion back into the patient, these engineered T cells recognize and eliminate malignant cells, leading to durable remissions in a subset of patients (8, 9). Despite its unprecedented success, not all patients respond favorably to CAR-T therapy (10, 11), highlighting the need for reliable prognostic markers to identify individuals likely to benefit from treatment. In this context, metabolic tumor volume (MTV) derived from positron emission tomography (PET) imaging has emerged as a promising biomarker for predicting treatment outcomes in NHL patients undergoing CAR-T therapy (12).

PET is a non-invasive imaging modality that provides functional information about tumor metabolism (13). By measuring the metabolic activity of tumors through the uptake of radiolabeled tracers, PET imaging enables the quantification of MTV, representing the total volume of metabolically active tumor tissue (13). High MTV levels have been associated with aggressive disease biology, treatment resistance, and poor prognosis in various cancer types, including NHL (14–16). Given its ability to capture the metabolic heterogeneity of tumors, MTV derived from PET holds potential as a prognostic biomarker for identifying patients at high risk of treatment failure or disease relapse following CAR-T therapy. Therefore, this meta-analysis aims to systematically evaluate the association between MTV and survival outcomes in NHL patients treated with CAR-T therapy, providing valuable insights into risk stratification and personalized treatment approaches in this patient population.

## Materials and methods

The Preferred Reporting Items for Systematic reviews and Meta-Analyses (PRISMA) statement (2020) (17, 18) was followed in this study. The Cochrane Handbook (19) for systematic review and meta-analysis was referenced throughout the study. The PRISMA Checklist of the meta-analysis is shown in **Supplementary Material 1**. The protocol of the study has been registered at the International Platform of Registered Systematic Review and Meta-analysis Protocols (INPLASY, https://inplasy.com/) with the registration number: INPLASY202450069.

### Literature search

Three electronic databases including PubMed, Web of Science, and Embase were used for literature search with a predefined combined search term including (1) “Chimeric Antigen Receptor” OR “Chimeric Antigen Receptors” OR “Chimeric T Cell Receptors” OR “Chimeric T-Cell Receptors” OR “Chimeric Antigen Receptor T Cell” OR “CAR-T” OR “Artificial T Cell Receptors” OR “Artificial T-Cell Receptors” OR “chimeric immunoreceptors” OR “Axicabtagene ciloleucel” OR “Axi-cel” OR “KTE-C19” OR “KTEC19” OR “CTL-019” OR “CTL019” OR “Yescarta” OR “Lisocabtagene” OR “maraleucel” OR “Liso-cel” OR “JCAR-017” OR “JCAR017” OR “Breyanzi” OR “Brexucabtagene” OR “autoleucel” OR “Brexu-cel” OR “KTE-X19” OR “KTEX19” OR “Tecartus” OR “Tisagenlecleucel” OR “Tisa-cel” OR “Kymriah” OR “ART-19” OR “CART19” OR “Axicabtagene” OR “ciloleucel” OR “Idecabtagene” OR “vicleucel” OR “Ciltacabtegene” OR “autoleucel”; (2) “lymphoma” OR “non-Hodgkin lymphoma”; (3) “18F-FDG PET/CT” OR “positron emission tomography” OR “positron emission tomography-computed tomography” OR “PET-CT” OR “PET” OR “PET CT” OR “PET/CT” OR “fluorodeoxyglucose” OR “metabolic tumor volume” OR “MTV”; and (4) “survival” OR “overall survival” OR “progression-free survival” OR “OS” OR “PFS” OR “death” OR “mortality” OR “progression” OR “prognosis” OR “cohort” OR “longitudinal” OR “prospective” OR “retrospective” OR “followed” OR “follow-up”. The detailed search strategy for each database is shown in **Supplementary Material 2**. Only studies with human subjects and published in peer-reviewed journals in English were included. A second-round check-up for the references of the relevant articles was also conducted. The final database search was achieved on April 1, 2024.

### Inclusion and exclusion criteria

Inclusion criteria:

(1) Observational studies with longitudinal follow-up published as full-length articles, such as cohort studies, nested case-control studies and *post-hoc* analysis of clinical trials;
(2) Studies involving adult patients with NHL who have not received CAR-T before inclusion;
(3) A PET scan (CT or MRI) was performed before CAR-T infusion, and MTV was derived from PET scan and analyzed as a categorized variable; the cutoff for defining a high versus a low MTV was consistent with the cutoffs used among the original studies;
(4) Compared the median progression-free survival (PFS) and/or overall survival (OS) after CAR-T treatment between NHL patients with a high versus a low MTV at baseline, and reported the hazard ratio (HR) and 95% confidence interval (CI) for the outcomes; or these data could be calculated or estimated from the original articles. For PFS, the outcome was defined as relapse, progression, all-cause deaths or the time of last follow-up, while for OS the outcome was defined as all-cause deaths or the time of last follow-up.

We excluded reviews, editorials, preclinical studies, cross-sectional studies, studies that included patients of Hodgkin lymphoma, without a PET scan at baseline, studies that did not measure MTV, studies of patients that did not receive CAR-T, or studies that did not report OS or PFS. In cases where there was potential overlap in patient population across multiple studies, only the study with the largest sample size was included in this analysis.

### Data collection and quality assessment

Two separate authors (LL and FJ) conducted a thorough search of academic literature, performed data collection and analysis, and independently assessed the quality of the studies. Any discrepancies that arose were resolved by involving the third author (HF) in discussion for final decision-making. Data on study information, design, patient characteristics including factors such as sample size, age, sex, diagnosis, Eastern Cooperative Oncology Group performance status (ECOG PS), medians of previous lines of therapy including transplant, lymphodepletion method, treatment information (type of CAR-T), imaging used for PET scan, timing of PET scan, methods to determine cutoff of MTV, cutoff value of MTV, median follow-up duration, survival outcomes (median PFS or OS), analytic model (univariate or multivariate), and whether the studies and the researchers were industry supported were extracted. The assessment of study quality was carried out using the Newcastle-Ottawa Scale (NOS) (20), which involved scoring based on criteria including participant selection process, comparability among groups, and validity of outcomes. This scale utilized a rating system ranging from 1 to 9 stars; higher stars indicated better study quality. The certainty of evidence was evaluated with the five Grading of Recommendations Assessment, Development, and Evaluation (GRADE) considerations of within- and across-study risk of bias (limitations in the study design and execution or methodological quality), inconsistency (or heterogeneity), indirectness of evidence, and imprecision of the effect estimates and risk of publication bias (21). A summary of findings table was made for the outcomes based on the Cochrane Handbook (19).

### Statistical methods

The association between baseline MTV and survival of patients with NHL after CAR-T therapy was presented as the HR and 95% CI compared between patients with a high versus low MTV before CAR-T infusion. Data of HRs and standard errors were calculated based on the 95% CIs or p values, followed by a logarithmical transformation to ensure stabilized variance and normalized distribution (19). The heterogeneity among studies was assessed using the Cochrane Q test and I^2^ statistic (22, 23), with I^2^ > 50% indicating significant statistical heterogeneity. In view of the differences of patient diagnosis, regimen of CAR-T, cutoff of MTV, and follow-up durations etc. across the included studies, significant clinical heterogeneity was deemed among these studies, and a random-effects model was used accordingly to incorporate the potential influence of heterogeneity (19). Sensitivity analysis involving exclusion of one study at a time was conducted to assess the robustness of findings (19). Subgroup analyses were performed to investigate if features such as study design, methods for determining the cutoff of MTV, analytic models, lines of previous treatment, and whether the study/researcher was industry supported could significantly affected the meta-analysis results (19). The univariate meta-regression analysis was performed to evaluate the potential influence of these variables on the outcomes, such as sample size, mean age, proportion of men, cutoff of MTV, follow-up duration, and study quality scores (19). Publication bias estimation involved constructing funnel plots initially evaluated through visual inspection for symmetricity before being analyzed using Egger’s regression test (24), where p < 0.05 indicates statistical significance. These analyses were conducted utilizing RevMan Version 5.1 from Cochrane Collaboration in Oxford, UK and Stata software version 12 from Stata Corporation in College Station, TX.

## Results

### Study inclusion

**Figure 1:**
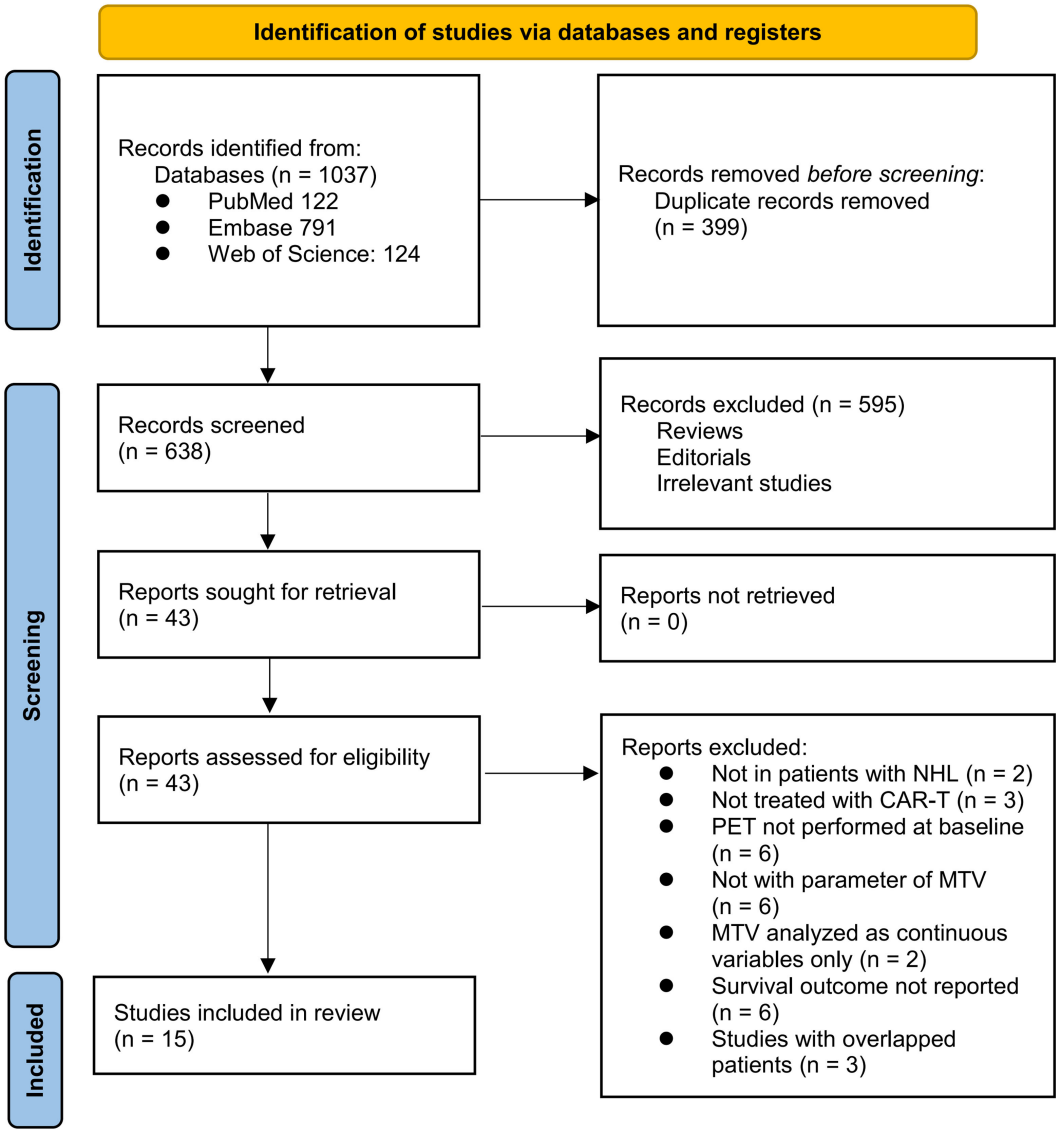
PRISMA flow diagram.

The process of selecting relevant studies for inclusion in the meta-analysis is depicted in **Figure 1**. Initially, 1037 potentially pertinent records were identified through thorough searches of three databases. Among these, 399 were removed due to duplication. Subsequent screening based on the titles and abstracts resulted in the exclusion of an additional 638 studies that did not align with the aim of the meta-analysis. The full texts of the remaining 43 records underwent independent review by two authors, leading to the removal of a further 28 studies for various reasons detailed in **Figure 1**. Ultimately, 15 observational studies remained and were considered suitable for subsequent quantitative analyses (25–39).

### Overview of the studies’ characteristics

**Tables 1**, **2** present the summarized characteristics of the included observational studies. Overall, four prospective studies (29, 30, 32, 33), 10 retrospective studies (25–28, 31, 34, 35, 37–39), and one *post-hoc* analysis (36) were included in the meta-analysis. Since one (26) of the included studies involved two independent cohorts of patients with NHL, these cohorts were included in the meta-analysis separately. These studies were published between 2019 and 2024, and performed in China, the United States, France, Sweden, Germany, Italy, Spain, and the Netherlands. All of the studies included patients with NHL, mostly of patients with relapsed or refractory large B-cell lymphoma. The sample size of the included study was generally small, varying from 16 to 175. The mean ages of the patients were 43 to 67 years, and the proportions of men varying between 44.0 to 76.9%. The median of previous lines of treatment was reported in seven studies, with 2 in one study (37), 3 in three studies (26, 31, 39), and 4 in another three studies (25, 28, 29). The methods of lymphodepletion were reported in eight studies, all of which were with fludarabine and cyclophosphamide (25–27, 29–32, 36). All of the included patients received the CAR-T treatment, with axicabtagene ciloleucel, tisagenlecleucel, brexucabtagene autoleucel, or lisocabtagene maraleucel. The PET scan was performed before CAR-T infusion, and the cutoff of MTV was determine by the medians of MTV in nine studies (25–30, 32, 33, 36), and via the Receiver Operating Characteristic (ROC) analysis in six studies (31, 34, 35, 37–39). The cutoff values for determining the high versus low MTV varied between 7.1 to 450 mL. The median follow-up duration was 7.7 to 42.6 months. The endpoint of PFS was reported in 15 cohorts (26–39), and the endpoint of OS was reported in 13 cohorts (25–27, 29–31, 33–35, 37–39). The univariate regression model was used in 11 cohorts (25, 27–30, 32, 34, 35, 37–39) in analyzing the association between MTV and survival outcome, while multivariate model was used in five cohorts (26, 31, 33, 36). Ten of the included studies or their researchers were industry supported (26, 28–33, 36, 38, 39), while five of them were not (25, 27, 34, 35, 37). The NOS of the included studies were five to nine stars, suggesting overall moderate to good study quality (**Table 3**).

**Table 1:** Patient and treatment characteristics of the included studies.

Author year	Country	Design	Diagnosis	Number of patients	Mean age (years)	Male (%)	ECOG PS	Previous lines of therapy (median)	Lymphodepletion method	CART-T treatment
Wang 2019 (25)	China	RC	NHL	19	43	63.2	NR	4	Fludarabine and Cy	Autologous anti-CD 19 CAR-T, trial based, containing 4–1BB domain
Dean 2020 C1 (26)	USA	RC	LBCL	48	63	65	0–3	3	Fludarabine and Cy	Axi-cel
Dean 2020 C2 (26)	USA	RC	LBCL	48	64	62.5	0–3	3	Fludarabine and Cy	Axi-cel
Zheng 2020 (27)	China	RC	R/R DLBCL	13	48	76.9	NR	NR	Fludarabine and Cy	Autologous anti-CD 19 CAR-T, commercial product, containing CD28 or 4–1BB domain
Sesques 2021 (28)	France	RC	Aggressive B-Cell lymphoma	72	60	61	0–1 (74%)	4	NR	Autologous anti-CD 19 CAR-T, commercial product, co-stimulatory domain not specified
Sjöholm 2022 (29)	Sweden	PC	R/R LBCL	16	63	44	0–2	4	Fludarabine and Cy	Autologous anti-CD 19 CAR-T (third generation), trial based, containing CD28 and 4–1BB domain
Winkelmann 2022 (30)	Germany	PC	R/R DLBCL and MCL	34	67	59	NR	NR	Fludarabine and Cy	Axi-cel, tisa-cel, brexu-cel, or liso-cel
Guidetti 2023 (32)	Italy	PC	R/R LBCL	47	55	68	0–1	NR	Fludarabine and Cy	Axi-cel or tisa-cel
Ligero 2023 (33)	Spain	PC	R/R LBCL	93	59	68	0 (34%)	NR	NR	Axi-cel or tisa-cel
Galtier 2023 (31)	France	RC	R/R LBCL	119	63	62	0–1 (83%)	3	Fludarabine and Cy	Axi-cel or tisa-cel
Zhou 2023 (34)	China	RC	DLBCL	61	52.1	60.7	0–1 (75.4%)	NR	NR	Autologous anti-CD 19 CAR-T, commercial product, co-stimulatory domain not specified
Locke 2024 (36)	USA, France, and the Netherlands	*Post-hoc*	R/R LBCL	175	58	61	NR	NR	Fludarabine and Cy	Axi-cel
Rojek 2024 (38)	USA	RC	R/R LBCL	61	66	70	0–3	NR	NR	Axi-cel, tisa-cel, or liso-cel
Gui 2024 (35)	China	RC	R/R DLBCL	38	55	61	0–2	NR	NR	Autologous anti-CD 19 CAR-T, commercial product, co-stimulatory domain not specified
Voltin 2024 (39)	Germany and Italy	RC	R/R LBCL	88	59	62.5	0–1 (71.6%)	3	NR	Axi-cel or tisa-cel
Marchal 2024 (37)	France	RC	LBCL	56	60.2	64	0–3	2	NR	Axi-cel or tisa-cel

CART-T, chimeric antigen receptor T cells; RC, retrospective cohort; PC, prospective cohort; NHL, Non-Hodgkin lymphoma; LBCL, large B-cell lymphoma; R/R, relapsed or refractory; DLBCL, diffuse large B cell lymphoma; MCL, mantle cell lymphoma; Axi-cel, axicabtagene ciloleucel; tisa-cel, tisagenlecleucel; brexu-cel, Brexucabtagene autoleucel; liso-cel, lisocabtagene maraleucel; ROC, receiver operating characteristic; ECOG PS, Eastern Cooperative Oncology Group performance status; NR, not reported; Cy, cyclophosphamide.

**Table 2:** PET imaging and follow-up characteristics of the included studies.

Author year	Imaging for PET	Timing of PET scan	MTV cutoff determination	MTV cutoff value (mL)	Follow-up duration (months)	Outcomes reported	Analytic model	Industry supported
Wang 2019 (25)	CT	Before CAR-T cell infusion	Median	72	12.5	OS	Univariate	No
Dean 2020 C1 (26)	CT	Before CAR-T cell infusion (median: 9 days)	Median	147.5	25	PFS and OS	Multivariate (Age, bridging therapy, and LDH before conditioning)	Yes
Dean 2020 C2 (26)	CT	Before CAR-T cell infusion (median: 11 days)	Median	147.5	12	PFS and OS	Multivariate (Age, bridging therapy, and LDH before conditioning)	Yes
Zheng 2020 (27)	CT	2 Weeks before CAR-T cell infusion	Median	64.9	7.7	PFS and OS	Univariate	No
Sesques 2021 (28)	CT	Before CAR-T cell infusion	Median	48.1	15	PFS	Univariate	Yes
Sjöholm 2022 (29)	MRI	Before CAR-T cell infusion	Median	39.5	42.6	PFS and OS	Univariate	Yes
Winkelmann 2022 (30)	CT	Within 2 Weeks before CAR-T cell infusion	Median	330	19.7	PFS and OS	Univariate	Yes
Guidetti 2023 (32)	CT	Before CAR-T cell infusion (median: 9 days)	Median	28	12	PFS	Univariate	Yes
Ligero 2023 (33)	CT	Before CAR-T cell infusion	Median	177	30	PFS and OS	Multivariate (Age, ECOG PS, lines of treatment, and costimulatory domain 4–1BB)	Yes
Galtier 2023 (31)	CT	Before CAR-T cell infusion	ROC curve analysis derived	80	12.6	PFS and OS	Multivariate (Age, elevated LDH, extranodal sites ≥2, and type of CAR-T)	Yes
Zhou 2023 (34)	CT	Before CAR-T cell infusion	ROC curve analysis derived	70	30	PFS and OS	Univariate	No
Locke 2024 (36)	CT	Before CAR-T cell infusion	Median	228.7	24.9	PFS	Multivariate (Age, LDH, and second line age-adjusted IPI)	Yes
Rojek 2024 (38)	NR	Before CAR-T cell infusion (median: 12 days)	ROC curve analysis derived	450	24	PFS and OS	Univariate	Yes
Gui 2024 (35)	CT	Before CAR-T cell infusion	ROC curve analysis derived	7.1	18.2	PFS and OS	Univariate	No
Voltin 2024 (39)	CT or MRI	Before CAR-T cell infusion	ROC curve analysis derived	259 for axi-cel and 11 for tisa-cel	17	PFS and OS	Univariate	Yes
Marchal 2024 (37)	CT	Before CAR-T cell infusion (median: 15 days)	ROC curve analysis derived	36	9.7	PFS and OS	Univariate	No

CART-T, chimeric antigen receptor T cells; PET, positron emission tomography; MTV, metabolic tumor volume; CT, computed tomography; MRI, magnetic resonance imaging; NR, not reported; ROC, receiver operating characteristic; OS, overall survival; PFS, progression-free survival; LDH, lactate dehydrogenase; ECOG PS, Eastern Cooperative Oncology Group performance status; IPI, International Prognostic Index.

**Table 3:** Study quality evaluation via the Newcastle-Ottawa Scale.

Study	Representativeness of the exposed cohort	Selection of the non-exposed cohort	Ascertainment of exposure	Outcome not present at baseline	Control for age	Control for other confounding factors	Assessment of outcome	Enough long follow-up duration	Adequacy of follow-up of cohorts	Total
Wang 2019 (25)	0	1	1	1	0	0	1	1	1	6
Dean 2020 C1 (26)	0	1	1	1	1	1	1	1	1	8
Dean 2020 C2 (26)	0	1	1	1	1	1	1	1	1	8
Zheng 2020 (27)	0	1	1	1	0	0	1	0	1	5
Sesques 2021 (28)	0	1	1	1	0	0	1	1	1	6
Sjöholm 2022 (29)	1	1	1	1	0	0	1	1	1	7
Winkelmann 2022 (30)	1	1	1	1	0	0	1	1	1	7
Guidetti 2023 (32)	1	1	1	1	0	0	1	1	1	7
Ligero 2023 (33)	1	1	1	1	1	1	1	1	1	9
Galtier 2023 (31)	1	1	1	1	1	1	1	1	1	9
Zhou 2023 (34)	0	1	1	1	0	0	1	1	1	6
Locke 2024 (36)	0	1	1	1	1	1	1	1	1	8
Rojek 2024 (38)	1	1	1	1	0	0	1	1	1	7
Gui 2024 (35)	0	1	1	1	0	0	1	1	1	6
Voltin 2024 (39)	0	1	1	1	0	0	1	1	1	6
Marchal 2024 (37)	0	1	1	1	0	0	1	0	1	5

### Meta-analysis for the association between MTV and PFS after CAR-T

**Figure 2:**
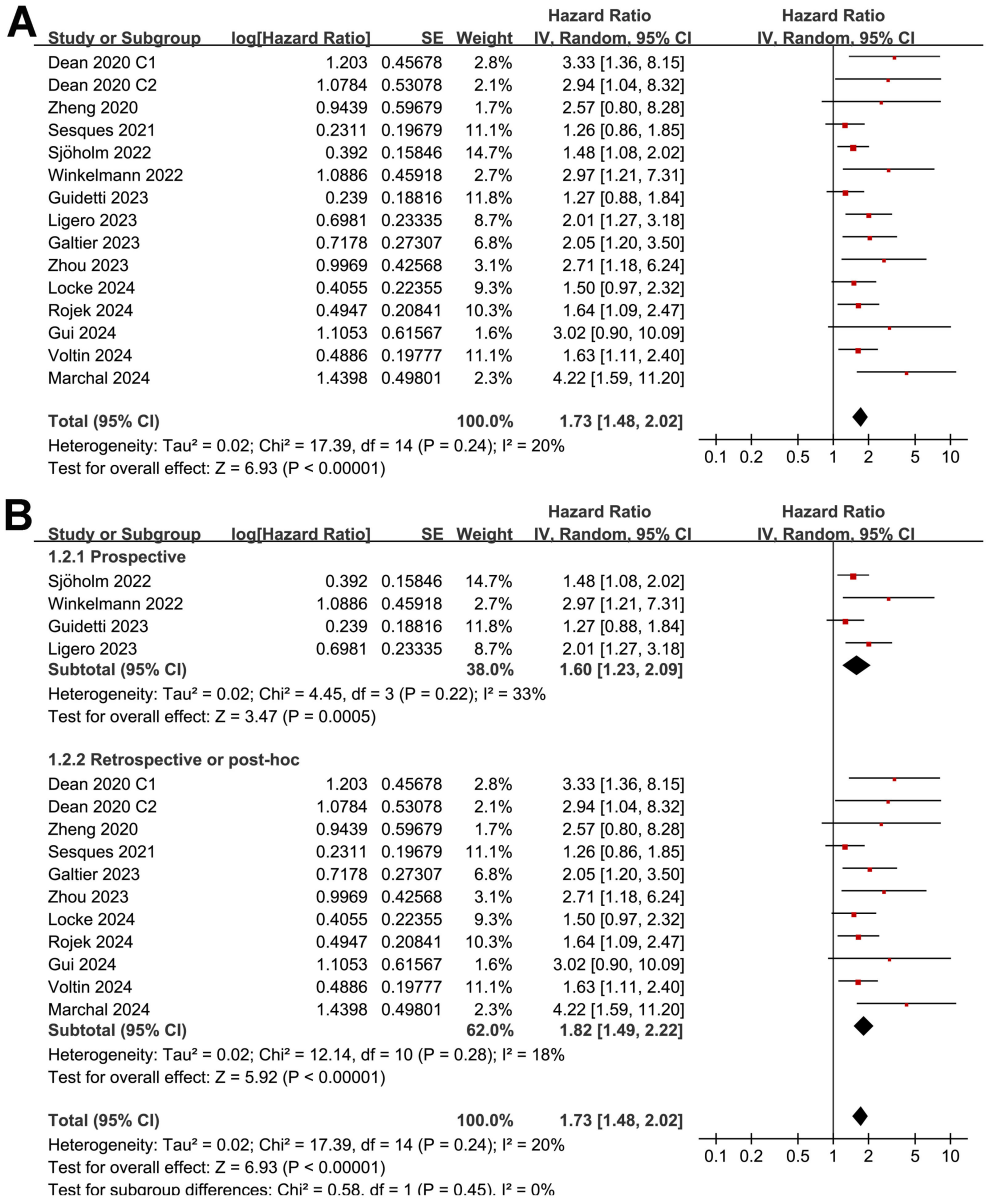
Forest plots for the meta-analysis of the association between MTV at baseline and PFS of NHL patients after CAR-T therapy; **(A)**, forest plots for the overall meta-analysis; and **(B)**, forest plots for the subgroup analysis according to study design.

Pooled results of 15 cohorts from 14 studies (26–39) suggested that compared to patients with a lower MTV at baseline, the NHL patients with a higher MTV before CAR-T infusion were associated with a poor PFS (HR: 1.73, 95% CI: 1.48 to 2.02, p < 0.001; I^2^ = 20%; **Figure 2A**) with a high certainty of evidence (**Table 4**). Results of the “leave-one-out” sensitivity analyses showed similar results (HR: 1.66 to 1.79, p all < 0.001; **Table 5**). In addition, sensitivity analysis limited to studies using lymphodepletion with fludarabine and cyclophosphamide showed similar results (HR: 1.71, 95% CI: 1.37 to 2.13, p < 0.001; I^2^ = 22%).

**Table 4:** Summary of findings.

Prognostic value of metabolic tumor volume for the survival of patients with Non-Hodgkin lymphoma treated with chimeric antigen receptor T cell therapy				
Patients: patients with Non-Hodgkin lymphoma treated with chimeric antigen receptor T cell therapy; Exposure: a high MTV on PET at enrollment; Comparison: a low MTV;				
Outcomes	Relative effect (95% CI)	Patient number (studies)	Certainty of the evidence (GRADE)	Comments
Progression-free survival	HR 1.73 (1.48 to 2.02)	969 (14 studies)	High	A high MTV on PET at enrollment is associated with a poor PFS in NHL patients after CAR-T treatment
Overall survival	HR 2.11 (1.54 to 2.89)	694 (12 studies)	Moderate^a^	A high MTV on PET at enrollment is likely to be associated with a poor OS in NHL patients after CAR-T treatment

GRADE Working Group grades of evidence; High certainty: We are very confident that the true effect lies close to that of the estimated effect. Moderate certainty: We are moderately confident in the estimated effect. The true effect is likely to be close to the estimated effect, but there is a possibility that it is substantially deferent. Low certainty: Our confidence in the estimated effect is limited: The true effect may be substantially different from the estimated effect. Very low certainty: We have very little confidence in the estimated effect. The true effect is likely to be substantially different from the estimated effect.

CI, confidence interval; HR hazard ratio; CART-T, chimeric antigen receptor T cells; PET, positron emission tomography; MTV, metabolic tumor volume; OS, overall survival; PFS, progression-free survival.

a Downgraded one point as inconsistency due to substantial heterogeneity.

**Table 5:** Sensitivity analysis by excluding one dataset at a time for the association between MTV and survival outcomes.

	Meta-analysis for the association between MTV and PFS			
Dataset omitted	HR [95% CI]	P for effect	I^2^	P for Cochrane Q test
Dean 2020 C1 (26)	1.68 [1.45, 1.95]	< 0.001	14%	0.30
Dean 2020 C2 (26)	1.71 [1.46, 1.99]	< 0.001	20%	0.24
Zheng 2020 (27)	1.72 [1.47, 2.02]	< 0.001	23%	0.21
Sesques 2021 (28)	1.78 [1.52, 2.08]	< 0.001	14%	0.30
Sjöholm 2022 (29)	1.79 [1.50, 2.13]	< 0.001	22%	0.22
Winkelmann 2022 (30)	1.69 [1.45, 1.98]	< 0.001	18%	0.26
Guidetti 2023 (32)	1.78 [1.52, 2.09]	< 0.001	13%	0.31
Ligero 2023 (33)	1.71 [1.45, 2.02]	< 0.001	22%	0.21
Galtier 2023 (31)	1.72 [1.46, 2.02]	< 0.001	23%	0.21
Zhou 2023 (34)	1.70 [1.45, 1.99]	< 0.001	19%	0.25
Locke 2024 (36)	1.77 [1.49, 2.10]	< 0.001	24%	0.19
Rojek 2024 (38)	1.76 [1.48, 2.09]	< 0.001	25%	0.18
Gui 2024 (35)	1.71 [1.47, 2.00]	< 0.001	21%	0.23
Voltin 2024 (39)	1.76 [1.48, 2.10]	< 0.001	25%	0.18
Marchal 2024 (37)	1.66 [1.44, 1.91]	< 0.001	6%	0.38

	Meta-analysis for the association between MTV and OS			
Dataset omitted	HR [95% CI]	P for effect	I^2^	P for Cochrane Q test
Wang 2019 (25)	2.20 [1.57, 3.09]	< 0.001	61%	0.003
Dean 2020 C1 (26)	1.98 [1.45, 2.70]	< 0.001	55%	0.01
Dean 2020 C2 (26)	2.02 [1.47, 2.76]	< 0.001	58%	0.006
Zheng 2020 (27)	2.05 [1.48, 2.82]	< 0.001	59%	0.005
Sjöholm 2022 (29)	2.29 [1.65, 3.19]	< 0.001	46%	0.04
Winkelmann 2022 (30)	2.18 [1.56, 3.05]	< 0.001	61%	0.003
Ligero 2023 (33)	2.23 [1.57, 3.17]	< 0.001	61%	0.003
Galtier 2023 (31)	1.80 [1.38, 2.35]	< 0.001	36%	0.10
Zhou 2023 (34)	2.04 [1.47, 2.83]	< 0.001	59%	0.005
Rojek 2024 (38)	2.21 [1.53, 3.20]	< 0.001	62%	0.003
Gui 2024 (35)	2.10 [1.53, 2.88]	< 0.001	61%	0.003
Voltin 2024 (39)	2.26 [1.58, 3.23]	< 0.001	61%	0.003
Marchal 2024 (37)	2.08 [1.49, 2.90]	< 0.001	60%	0.003

MTV, metabolic tumor volume; PFS, progression-free survival; OS, overall survival; HR, hazard ratio; CI, confidence interval.

**Figure 3:**
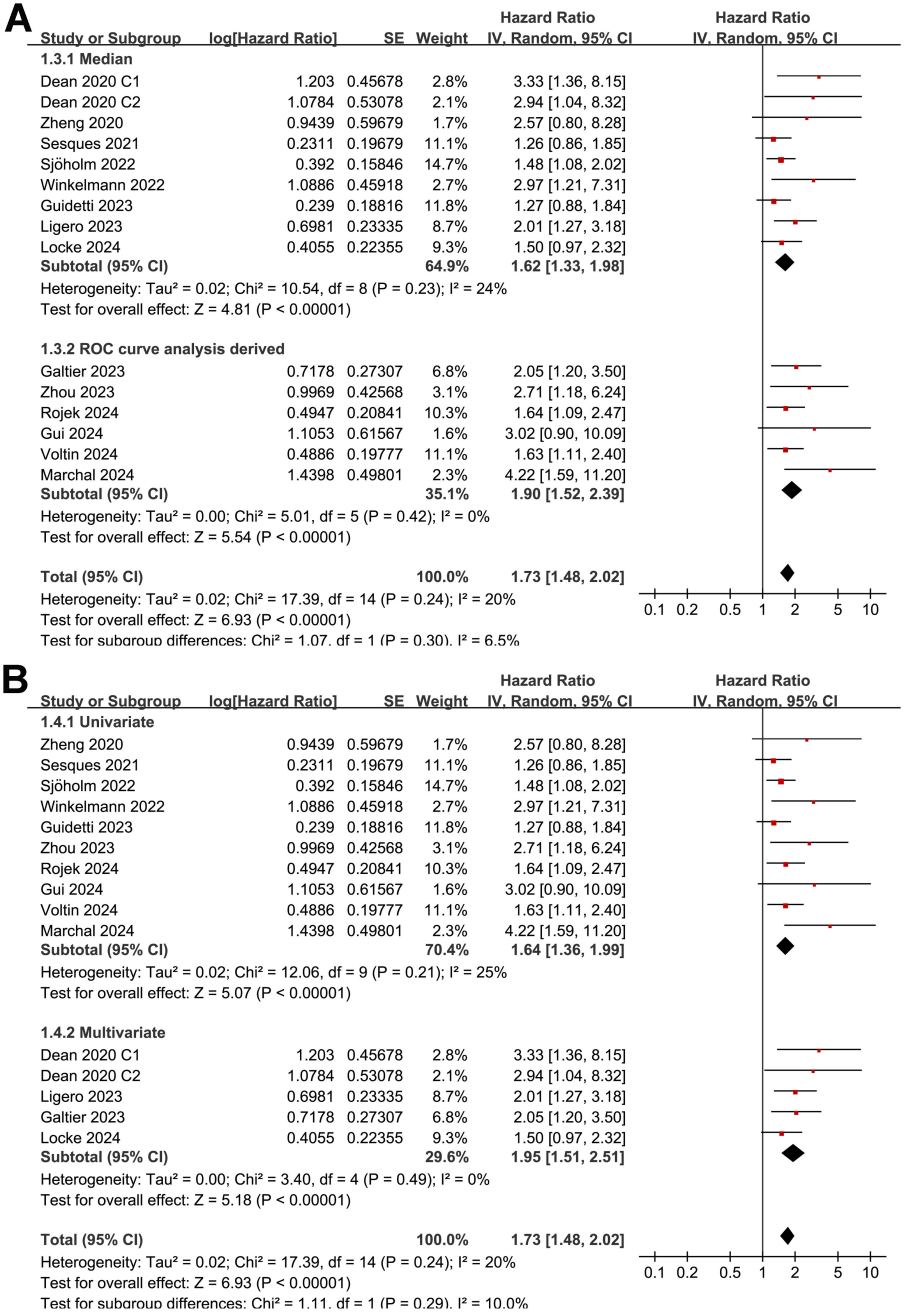
Forest plots for the subgroup analysis of the association between MTV at baseline and PFS of NHL patients after CAR-T therapy; **(A)**, forest plots for the subgroup analysis according to the methods for determining the cutoff of MTV; and **(B)**, forest plots for the subgroup analysis according to the analytic models.

**Figure 4:**
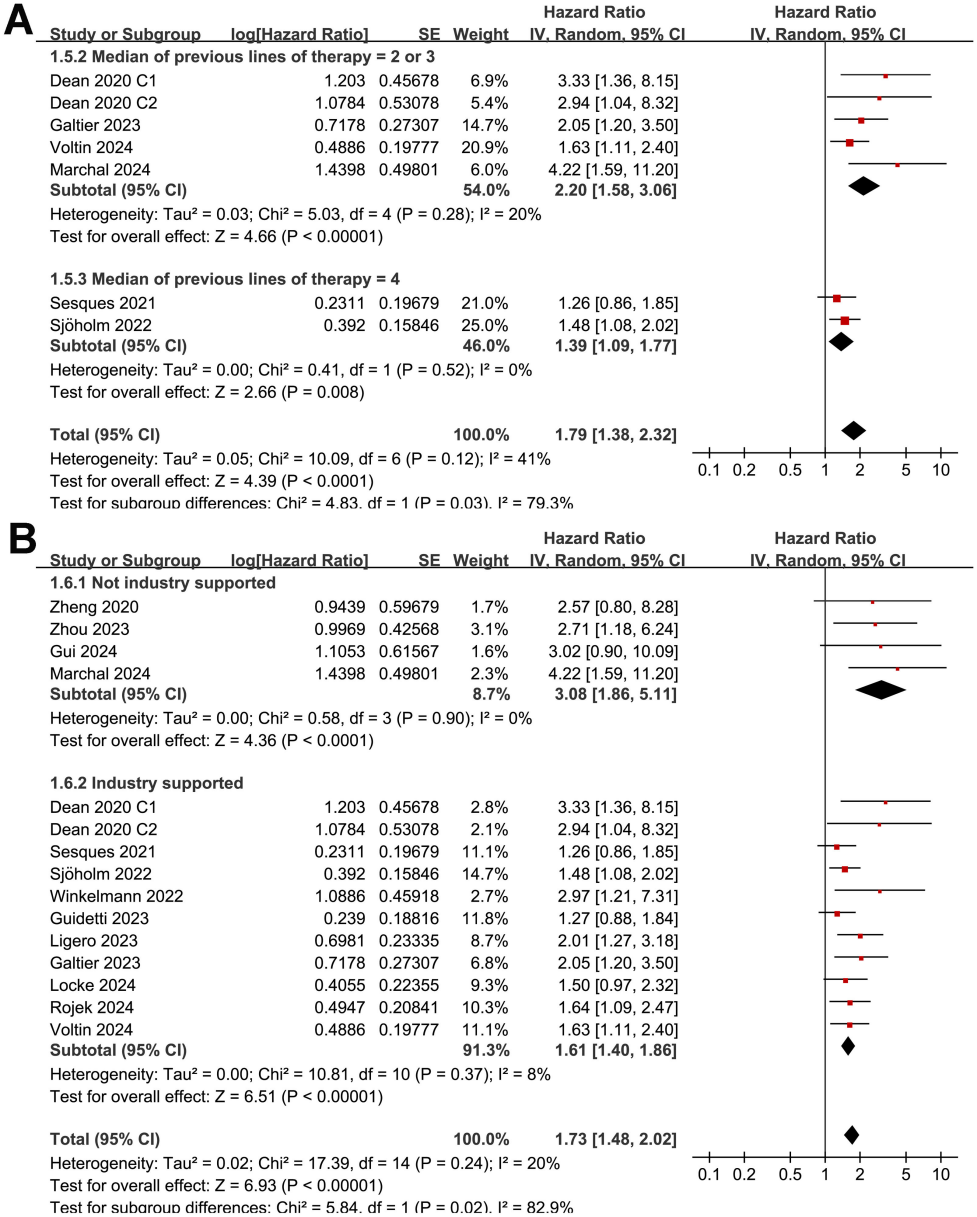
Forest plots for the subgroup analysis of the association between MTV at baseline and PFS of NHL patients after CAR-T therapy; **(A)**, forest plots for the subgroup analysis according to the median of previous lines of treatments; and **(B)**, forest plots for the subgroup analysis according to whether the study was industry supported.

The subgroup analysis showed that the association between MTV and the PFS of NHL patients after CAR-T was consistent in prospective and retrospective/*post-hoc* studies (HR: 1.60 versus 1.82, p for subgroup difference = 0.45; **Figure 2B**), in studies with cutoff of MTV determined by the median or ROC curve analysis (HR: 1.62 versus 1.90, p for subgroup difference = 0.30; **Figure 3A**), and in studies with univariate and multivariate analyses (HR: 1.64 versus 1.95, p for subgroup difference = 0.29; **Figure 3B**). Interestingly, a stronger association between MTV and PFS was observed for patients with median of lines of previous treatment of 2 or 3 as compared to those of 4 (HR: 2.20 versus 1.39, p for subgroup difference = 0.03; **Figure 4A**). In addition, a stronger association between MTV and PFS was also observed in non-industry supported studies as compared to industry supported studies (HR: 3.08 versus 1.61, p for subgroup difference = 0.02; **Figure 4B**).

Further meta-regression analyses suggested that the association between MTV and PFS of NHL patients after CAR-T was not significantly affected by study sample size, mean age, proportion of men, cutoff value of MTV, follow-up duration, or study quality scores (**Table 6**, p all > 0.05).

**Table 6:** Results of univariate meta-regression analysis.

Variables	HR for PFS			HR for OS		
	Coefficient	95% CI	P values	Coefficient	95% CI	P values
Sample size	-0.00055	-0.00475 to 0.00365	0.78	-0.0012	-0.0142 to 0.0118	0.84
Mean age (years)	0.0071	-0.0376 to 0.0519	0.74	0.00078	-0.05747 to 0.05902	0.98
Men (%)	0.0061	-0.0159 to 0.0282	0.56	0.022	-0.022 to 0.066	0.29
Cutoff of MTV (mL)	0.00034	-0.00098 to 0.00166	0.59	-0.00076	-0.00368 to 0.00217	0.58
Follow-up duration (months)	-0.0025	-0.0203 to.01534	0.77	-0.023	-0.053 to 0.006	0.10
NOS	0.035	-0.126 to 0.196	0.65	0.078	-0.214 to 0.371	0.57

HR, hazard ratio; PFS, progression-free survival; OS, overall survival; CI, confidence interval; MTV, metabolic tumor volume; NOS, Newcastle-Ottawa Scale.

### Meta-analysis for the association between MTV and OS after CAR-T

**Figure 5:**
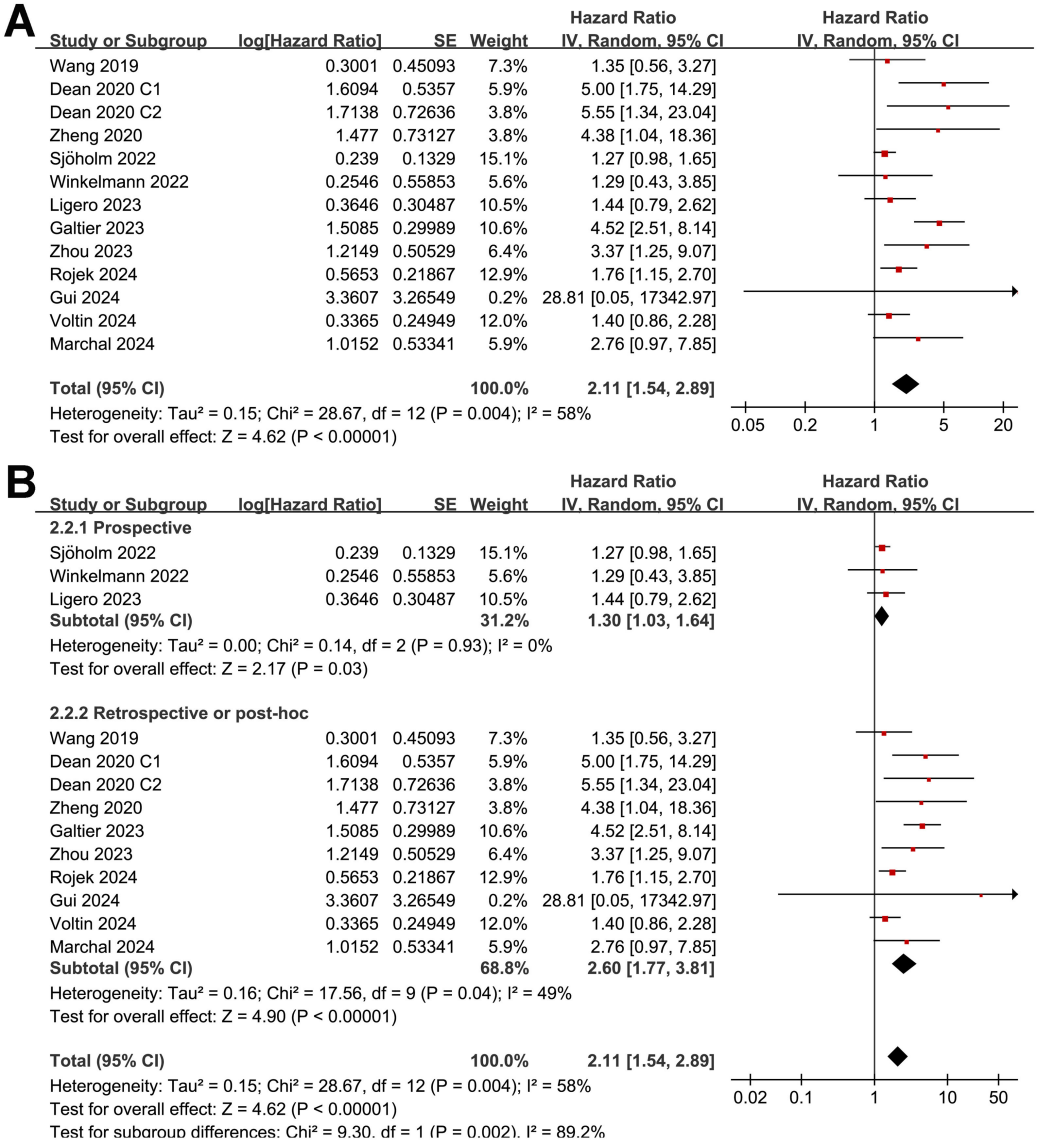
Forest plots for the meta-analysis of the association between MTV at baseline and OS of NHL patients after CAR-T therapy; **(A)**, forest plots for the overall meta-analysis; and **(B)**, forest plots for the subgroup analysis according to study design.

Synthesized results of 11 cohorts (25, 27–30, 32, 34, 35, 37–39) suggested a potential association between a high MTV at baseline and the poor OS of patients after CAR-T therapy (HR: 2.11, 95% CI: 1.54 to 2.89, p < 0.001; I^2^ = 58%; **Figure 5A**) with a moderate certainty of evidence (**Table 4**). The results of the “leave-one-out” sensitivity analyses further confirmed the robustness of the finding (HR: 1.80 to 2.29, p all < 0.05; **Table 5**). Additionally, sensitivity analysis limited to studies using lymphodepletion with fludarabine and cyclophosphamide showed similar results (HR: 2.53, 95% CI: 1.39 to 4.64, p = 0.003; I^2^ = 75%).

**Figure 6:**
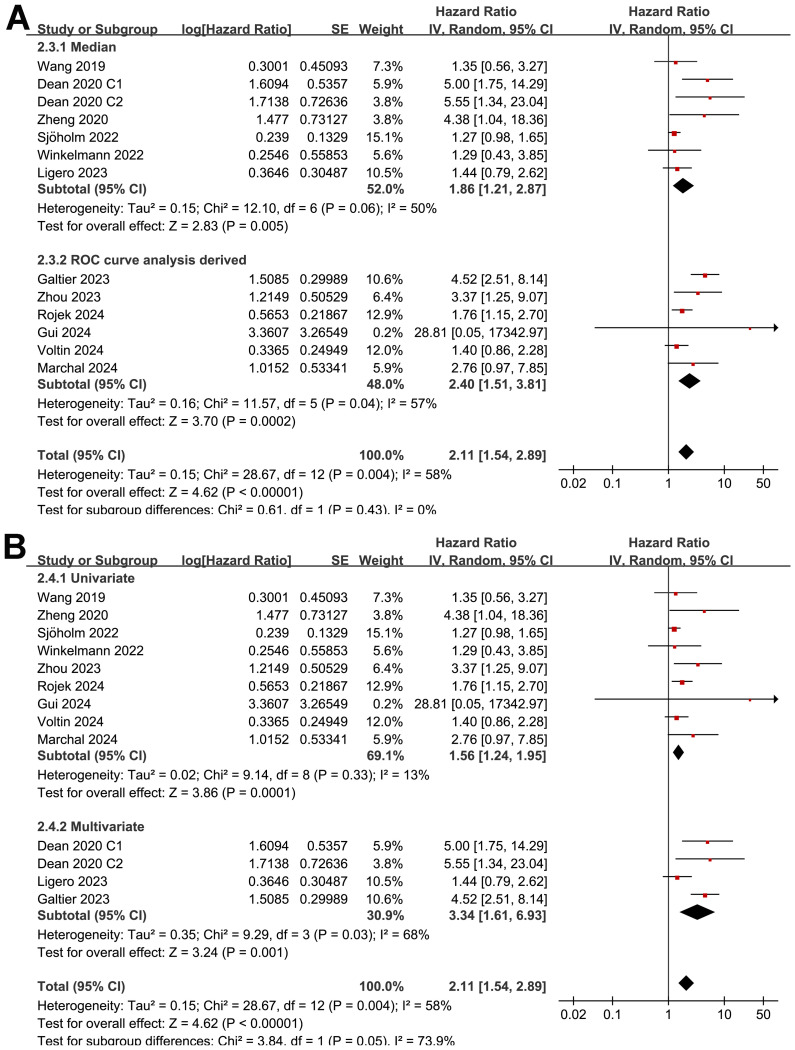
Forest plots for the subgroup analysis of the association between MTV at baseline and OS of NHL patients after CAR-T therapy; **(A)**, forest plots for the subgroup analysis according to the methods for determining the cutoff of MTV; and **(B)**, forest plots for the subgroup analysis according to the analytic models.

**Figure 7:**
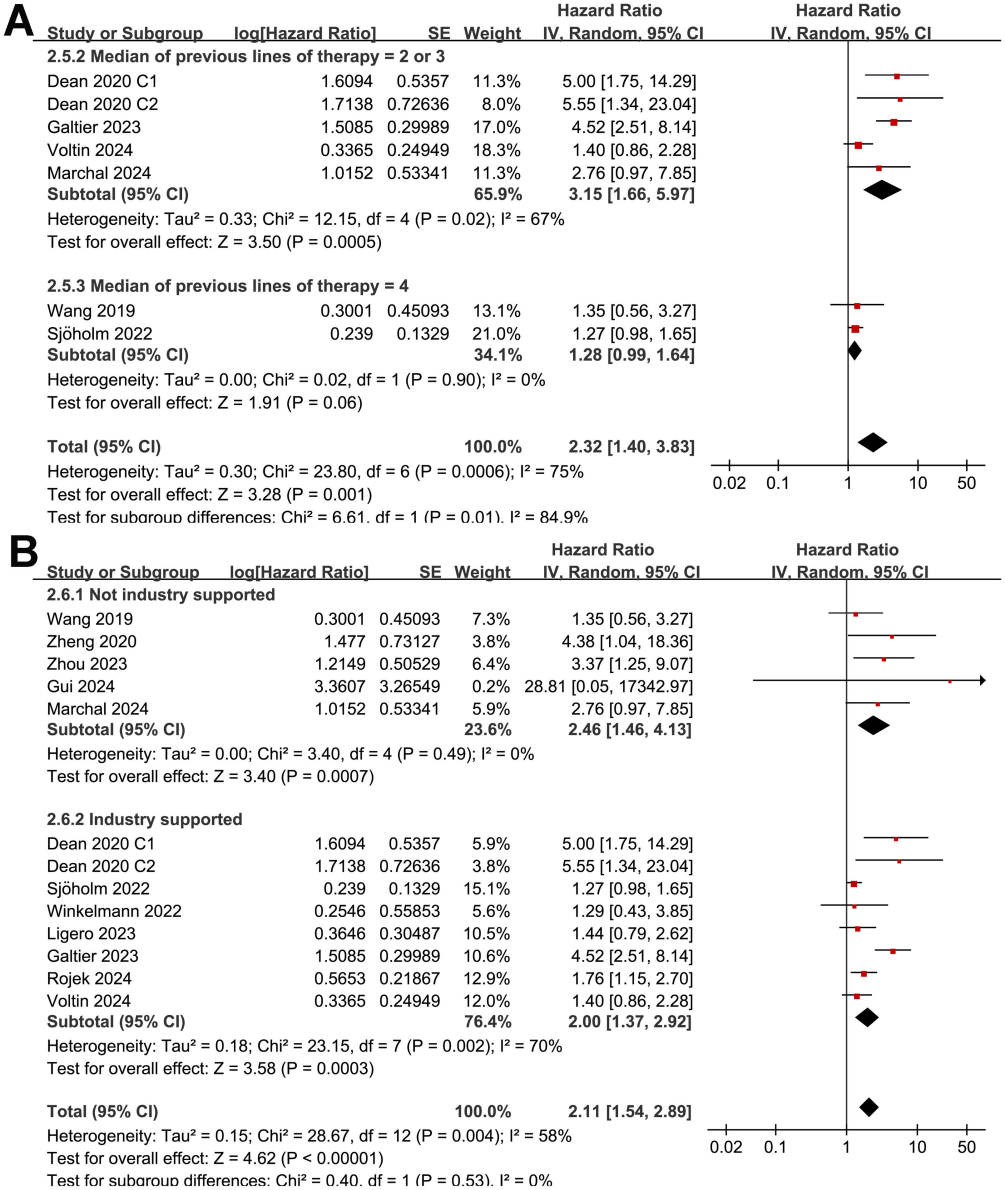
Forest plots for the subgroup analysis of the association between MTV at baseline and OS of NHL patients after CAR-T therapy; **(A)**, forest plots for the subgroup analysis according to the median of previous lines of treatments; and **(B)**, forest plots for the subgroup analysis according to whether the study was industry supported.

Although the results were both statistically significant, the subgroup analysis suggested a stronger association between MTV and poor OS in retrospective/*post-hoc* studies than in the prospective studies (HR: 2.60 versus 1.30, p for subgroup difference = 0.002; **Figure 5B**). Further subgroup analyses showed that the association between a high MTV and poor OS was not significantly affected by methods for defining the cutoff of MTV (HR: 1.86 versus 2.40, p for subgroup difference = 0.43; **Figure 6A**) or the analytic models (HR: 1.56 versus 3.34, p for subgroup difference = 0.05; **Figure 6B**). Interestingly, a stronger association between MTV and OS was observed for patients with median of lines of previous treatment of 2 or 3 as compared to those of 4 (HR: 3.15 versus 1.28, p for subgroup difference = 0.03; **Figure 7A**). A similar association between MTV and OS was observed in non-industry supported and industry supported studies (HR: 2.46 versus 2.00, p for subgroup difference = 0.53; **Figure 7B**).

The results of the meta-regression analysis did not suggest the association between a high MTV and poor OS was significantly modified by the study sample size, mean age, proportion of men, cutoff value of MTV, follow-up duration, or study quality scores (**Table 6**, p all > 0.05).

### Publication bias evaluation

**Figure 8:**
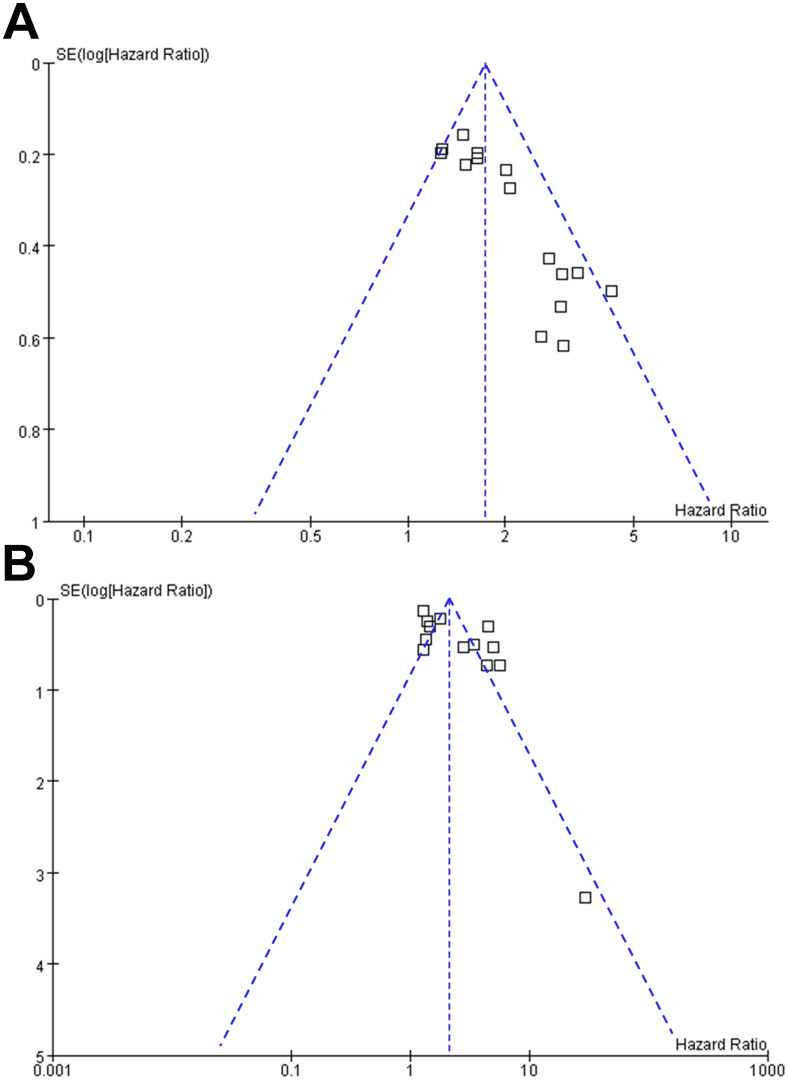
Funnel plots for the publication bias underlying the meta-analyses of the association between MTV at baseline and survival of NHL patients after CAR-T therapy; **(A)**, funnel plots for the outcome of PFS; and **(B)**, funnel plots for the outcome of OS.

Funnel plots in **Figures 8A, B** display the meta-analyses of the relationships between MTV at baseline with PFS and OS in NHL patients after CAR-T therapy. The symmetrical nature of the funnel plots indicates a low likelihood of publication biases. Additionally, Egger’s regression test results also suggest a low risk of publication bias (p = 0.13 for the outcome of PFS and 0.25 for the outcome of OS).

## Discussion

The findings of this meta-analysis confirmed the prognostic value of MTV derived from PET in patients with NHL undergoing CAR-T therapy. The pooled results from 15 observational studies demonstrate a consistent association between higher baseline MTV and poorer survival outcomes, including PFS and OS following CAR-T therapy. Interestingly, subgroup analysis suggested a stronger association between MTV and poor OS/PFS in patients with median of lines of previous treatment of 2 or 3 as compared to those with 4. In addition, subgroup analyses revealed that this association remained robust across different study designs, methods for determining MTV cutoffs, and analytical models, suggesting the reliability and generalizability of the findings. Furthermore, meta-regression analyses indicated that various factors, including sample size, patient demographics, MTV cutoff values, and study quality, did not significantly influence the observed association between MTV and survival, reinforcing the validity of the results.

The association between high MTV and poor survival outcomes in NHL patients after CAR-T therapy might be attributed to several potential mechanisms. Firstly, elevated MTV reflects a higher tumor burden and metabolic activity, indicating more aggressive disease biology and increased resistance to therapy (40). Secondly, tumors with high metabolic activity may exhibit greater heterogeneity and genomic instability, leading to treatment resistance and disease relapse (41). Moreover, the tumor microenvironment characterized by metabolic dysregulation, hypoxia, and immune evasion may contribute to therapeutic resistance and tumor progression (42). In addition, a high MTV has been related to an increased risk of CAR-T related toxicity in patients with NHL, such as the risk of grade 3+ immune effector cell-associated neurotoxicity syndrome (43, 44), which may also be an important reason for the reduced OS in these patients. Understanding these underlying mechanisms can guide the development of novel therapeutic strategies targeting metabolic vulnerabilities or enhancing CAR-T cell efficacy in high-MTV tumors.

Results of subgroup analysis findings showed a stronger association between MTV and poor OS/PFS in patients with median of lines of previous treatment of 2 or 3 as compared to those with 4, which suggests that the prognostic value of MTV on PET scans in patients with NHL undergoing CAR T-cell therapy might be modulated by the number of previous treatment lines. However, these findings suggest that patients with fewer lines of treatment may have a higher risk of poor outcome (PFS/OS), which seems paradoxical considering patients with fewer lines of treatment are expected to have fitter T-cells (45, 46) and better treatment outcomes after CAR-T therapy. It seems that there are other confounding factors accounting for these results, such as potentially increased rate of CAR-T toxicities or infections in patients with fewer lines of treatment, which were not assessed in this meta-analysis. Besides, meta-analysis for the outcomes of PFS and OS analysis showed moderate and high heterogeneity, respectively. Therefore the results should be interpreted very cautiously. Moreover, for the subgroups with the median of previous lines treatment of 4, very few studies (only 2) were available, which probably is too small to capture the true effect. Additionally, separate sub-analysis on the CAR-T product was not undertaken, which may also have an impact on the outcomes. For example, the study with the third generation CAR has been included in the analysis (29) and currently there is not strong evidence on how these products perform and their impact on outcomes, especially compared with the licensed second generation ones. Accordingly, future prospective studies with large sample size are still needed to determine whether previous lines of treatment may modify the association between MTV and survival outcomes of NHL patients after CAR-T therapy, and to explore the potential underlying mechanisms.

The strengths of this meta-analysis include its comprehensive literature search, rigorous inclusion criteria, and robust statistical analyses accounting for heterogeneity across studies. However, certain limitations should be acknowledged. First, 11 retrospective or *post-hoc* analysis was included in the meta-analysis, which may expose the results to the risk of recall and selection biases. However, subgroup analysis according to study design showed consistent results. Second, 13 of the included studies enrolled patients with LBC, and the results of the meta-analysis were mostly driven by studies with patients of LBCL. The association between MTV and the survival of patients with other subtypes of NHL should still be investigated in the future. Third, moderately statistical heterogeneity was observed for the meta-analysis of the association between MTV and OS of NHL patients after CAR-T therapy, and the subgroup analysis results based on limited study-level data suggest that the number of previous treatment lines may modulate the association. We were unable to determine the influence of patient physical status on the association between MTV and survival outcomes in a subgroup or meta-regression analysis because none of the included studies reported the outcome according to the class of ECOG PS, and on study-level, data of ECOG PS of the included studies were reported in a non-uniform manner. In addition, recent publications have correlated a high tumor burden (reflected by MTV) with a higher risk of infections, and the latter has been related with a higher non-relapse mortality in the long-term which may impact the survival outcomes (12, 47). However, only one of the included studies reported the incidence of infection of the included patients (38). Future studies are needed to determine if the incidence of infection after CAR-T may modify the association between MTV and survival outcomes of patients with NHL. Finally, the univariate-regression analysis was used in 11 of the included studies. Although a consistent result was observed in subgroup analysis of multivariate analysis, we could not exclude the possibility that residual unadjusted factors may confound the association between a high baseline MTV and poor survival of NHL patients after CAR-T therapy.

MTV offers several advantages in the assessment of NHL patients undergoing CAR-T therapy. Firstly, it provides a quantitative measure of tumor burden, enabling a comprehensive evaluation beyond conventional anatomical imaging modalities. This quantitative assessment facilitates early detection of disease progression or response to therapy, guiding timely adjustments in treatment strategies. Additionally, high baseline MTV serves as a prognostic biomarker, aiding in risk stratification and identification of patients at higher risk of treatment failure or disease relapse. Despite its potential advantages, the measurement of MTV presents several methodological challenges that need to be addressed (48, 49). Variability in PET imaging protocols, including tracer dose and uptake time, can impact the accuracy and reproducibility of MTV measurements (48). Standardization of imaging protocols across institutions is crucial to ensure consistency and comparability of MTV values. Furthermore, accurate delineation of tumor boundaries on PET images is challenging, particularly in cases of diffuse or heterogeneous disease involvement (48, 49). Standardized segmentation algorithms and quality assurance measures are needed to improve reproducibility. Moreover, PET/CT fusion artifacts and optimal thresholding methods for defining metabolically active tumor voxels pose additional challenges to MTV measurement (48). Advanced image registration techniques and standardized thresholding algorithms are required to mitigate artifacts and improve the reliability of MTV quantification (48). Despite these challenges, addressing methodological considerations in MTV measurement will enhance its utility as a prognostic biomarker and guide clinical decision-making in the management of NHL patients undergoing CAR-T therapy.

From a clinical perspective, the identification of high MTV as a predictor of poor survival outcomes in NHL patients treated with CAR-T therapy has important implications for risk stratification, treatment selection, and patient management. Incorporating MTV assessment into routine clinical practice may facilitate personalized treatment approaches, such as intensification of therapy or consideration of alternative treatment strategies in patients with high-risk disease. Furthermore, future studies should focus on validating these findings in prospective cohorts, elucidating the biological mechanisms underlying the association between MTV and survival, and exploring therapeutic strategies to overcome treatment resistance in high-MTV tumors.

## Conclusions

In conclusion, this meta-analysis provides pilot evidence for the prognostic significance of MTV in NHL patients undergoing CAR-T therapy. Higher baseline MTV is consistently associated with poorer survival outcomes, highlighting its potential utility as a predictive biomarker for risk stratification and treatment optimization in this patient population. Further research efforts are warranted to validate these findings, elucidate underlying mechanisms, and translate these insights into clinical practice to improve outcomes for NHL patients undergoing CAR-T therapy.

## Data Availability

The original contributions presented in the study are included in the article/**Supplementary Material**. Further inquiries can be directed to the corresponding authors.

## References

[1] Siegel RL Giaquinto AN Jemal A . Cancer statistics, 2024. CA Cancer J Clin. (2024) 74:12–49. doi: 10.3322/caac.21820 38230766

[2] Shankland KR Armitage JO Hancock BW . Non-hodgkin lymphoma. Lancet. (2012) 380:848–57. doi: 10.1016/S0140-6736(12)60605-9 22835603

[3] Miranda-Filho A Pineros M Znaor A Marcos-Gragera R Steliarova-Foucher E Bray F . Global patterns and trends in the incidence of non-Hodgkin lymphoma. Cancer Causes Control. (2019) 30:489–99. doi: 10.1007/s10552-019-01155-5 30895415

[4] Zhang N Wu J Wang Q Liang Y Li X Chen G . Global burden of hematologic Malignancies and evolution patterns over the past 30 years. Blood Cancer J. (2023) 13:82. doi: 10.1038/s41408-023-00853-3 37193689 PMC10188596

[5] Ansell SM . Non-hodgkin lymphoma: diagnosis and treatment. Mayo Clin Proc. (2015) 90:1152–63. doi: 10.1016/j.mayocp.2015.04.025 26250731

[6] Pang Y Ghosh N . Novel and multiple targets for chimeric antigen receptor-based therapies in lymphoma. Front Oncol. (2024) 14:1396395. doi: 10.3389/fonc.2024.1396395 38711850 PMC11070555

[7] Giraudo MF Jackson Z Das I Abiona OM Wald DN . Chimeric antigen receptor (CAR)-T cell therapy for non-hodgkin’s lymphoma. Pathog Immun. (2024) 9:1–17. doi: 10.20411/pai.v9i1 PMC10972674 38550613

[8] June CH Sadelain M . Chimeric antigen receptor therapy. N Engl J Med. (2018) 379:64–73. doi: 10.1056/NEJMra1706169 29972754 PMC7433347

[9] Abbasi S Totmaj MA Abbasi M Hajazimian S Goleij P Behroozi J . Chimeric antigen receptor T (CAR-T) cells: Novel cell therapy for hematological Malignancies. Cancer Med. (2023) 12:7844–58. doi: 10.1002/cam4.5551 PMC10134288 36583504

[10] Cappell KM Sherry RM Yang JC Goff SL Vanasse DA McIntyre L . Long-term follow-up of anti-CD19 chimeric antigen receptor T-cell therapy. J Clin Oncol. (2020) 38:3805–15. doi: 10.1200/JCO.20.01467 PMC7655016 33021872

[11] Jacobson CA Munoz J Sun F Kanters S Limbrick-Oldfield EH Spooner C . Real-world outcomes with chimeric antigen receptor T cell therapies in large B cell lymphoma: A systematic review and meta-analysis. Transplant Cell Ther. (2024) 30:77.e1– e15. doi: 10.1016/j.jtct.2023.10.017 37890589

[12] Linguanti F Abenavoli EM Berti V Lopci E . Metabolic imaging in B-cell lymphomas during CAR-T cell therapy. Cancers (Basel). (2022) 14. doi: 10.3390/cancers14194700 PMC9562671 36230629

[13] Murad V Kohan A Ortega C Prica A Veit-Haibach P Metser U . Role of FDG PET/CT in patients with lymphoma treated with chimeric antigen receptor T-cell therapy: current concepts. AJR Am J Roentgenol. (2024) 222:e2330301. doi: 10.2214/AJR.23.30301 38054958

[14] Barrington SF . Advances in positron emission tomography and radiomics. Hematol Oncol. (2023) 41 Suppl 1:11–9. 10.1002/hon.3137 PMC10775708 37294959

[15] Pellegrino S Fonti R Pulcrano A Del Vecchio S . PET-based volumetric biomarkers for risk stratification of non-small cell lung cancer patients. Diagnostics (Basel). (2021) 11. doi: 10.3390/diagnostics11020210 PMC7911597 33573333

[16] Tutino F Giovannini E Pastorino S Ferrando O Giovacchini G Ciarmiello A . Methodological aspects and the prognostic value of metabolic tumor volume assessed with 18F-FDG PET/CT in lymphomas. Curr Radiopharm. (2022) 15:259–70. doi: 10.2174/1874471015666220329120631 35352655

[17] Page MJ McKenzie JE Bossuyt PM Boutron I Hoffmann TC Mulrow CD . The PRISMA 2020 statement: an updated guideline for reporting systematic reviews. BMJ. (2021) 372:n71. 33782057 10.1136/bmj.n71 PMC8005924

[18] Page MJ Moher D Bossuyt PM Boutron I Hoffmann TC Mulrow CD . PRISMA 2020 explanation and elaboration: updated guidance and exemplars for reporting systematic reviews. BMJ. (2021) 372:n160. doi: 10.1136/bmj.n160 33781993 PMC8005925

[19] Higgins J Thomas J Chandler J Cumpston M Li T Page M . Cochrane Handbook for Systematic Reviews of Interventions version 6.2. London, UK: The Cochrane Collaboration (2021). Available at: www.training.cochrane.org/handbook.

[20] Wells GA Shea B O’Connell D Peterson J Welch V Losos M . The Newcastle-Ottawa Scale (NOS) for assessing the quality of nonrandomised studies in meta-analyses (2010). Available online at: http://www.ohri.ca/programs/clinical_epidemiology/oxford.asp (Accessed April 20, 2024).

[21] Guyatt G Oxman AD Akl EA Kunz R Vist G Brozek J . GRADE guidelines: 1. Introduction-GRADE evidence profiles and summary of findings tables. J Clin Epidemiol. (2011) 64:383–94. doi: 10.1016/j.jclinepi.2010.04.026 21195583

[22] Higgins JP Thompson SG . Quantifying heterogeneity in a meta-analysis. Stat Med. (2002) 21:1539–58. doi: 10.1002/sim.1186 12111919

[23] Patsopoulos NA Evangelou E Ioannidis JP . Sensitivity of between-study heterogeneity in meta-analysis: proposed metrics and empirical evaluation. Int J Epidemiol. (2008) 37:1148–57. doi: 10.1093/ije/dyn065 PMC6281381 18424475

[24] Egger M Davey Smith G Schneider M Minder C . Bias in meta-analysis detected by a simple, graphical test. BMJ. (1997) 315:629–34. doi: 10.1136/bmj.315.7109.629 PMC2127453 9310563

[25] Wang J Hu Y Yang S Wei G Zhao X Wu W . Role of fluorodeoxyglucose positron emission tomography/computed tomography in predicting the adverse effects of chimeric antigen receptor T cell therapy in patients with non-hodgkin lymphoma. Biol Blood Marrow Transplant. (2019) 25:1092–8. doi: 10.1016/j.bbmt.2019.02.008 30769193

[26] Dean EA Mhaskar RS Lu H Mousa MS Krivenko GS Lazaryan A . High metabolic tumor volume is associated with decreased efficacy of axicabtagene ciloleucel in large B-cell lymphoma. Blood Adv. (2020) 4:3268–76. doi: 10.1182/bloodadvances.2020001900 PMC7391155 32702097

[27] Zheng XQ Ding CY Zou YX Zhu HY Wang L Fan L . Roles of PET/CT in predicting the prognosis of diffuse large B cell lymphoma patients treated with chimeric antigen receptor T cell therapy. Chin J Exp Hematol. (2020) 28:1189–96. 10.19746/j.cnki.issn.1009-2137.2020.04.018 32798397

[28] Sesques P Tordo J Ferrant E Safar V Wallet F Dhomps A . Prognostic impact of 18F-FDG PET/CT in patients with aggressive B-cell lymphoma treated with anti-CD19 chimeric antigen receptor T cells. Clin Nucl Med. (2021) 46:627–34. doi: 10.1097/RLU.0000000000003756 34115706

[29] Sjoholm T Korenyushkin A Gammelgard G Saren T Lovgren T Loskog A . Whole body FDG PET/MR for progression free and overall survival prediction in patients with relapsed/refractory large B-cell lymphomas undergoing CAR T-cell therapy. Cancer Imaging. (2022) 22:76. doi: 10.1186/s40644-022-00513-y 36575477 PMC9793670

[30] Winkelmann M Bucklein VL Blumenberg V Rejeski K Ruzicka M Unterrainer M . Lymphoma tumor burden before chimeric antigen receptor T-Cell treatment: RECIL vs. Lugano vs. metabolic tumor assessment. Front Oncol. (2022) 12:974029. doi: 10.3389/fonc.2022.974029 36158658 PMC9492918

[31] Galtier J Vercellino L Chartier L Olivier P Tabouret-Viaud C Mesguich C . Positron emission tomography-imaging assessment for guiding strategy in patients with relapsed/refractory large B-cell lymphoma receiving CAR T cells. Haematologica. (2023) 108:171–80. doi: 10.3324/haematol.2021.280550 PMC9827160 35678029

[32] Guidetti A Dodero A Lorenzoni A Pizzamiglio S Argiroffi G Chiappella A . Combination of Deauville score and quantitative positron emission tomography parameters as a predictive tool of anti-CD19 chimeric antigen receptor T-cell efficacy. Cancer. (2023) 129:255–63. doi: 10.1002/cncr.34532 PMC10099560 36385707

[33] Ligero M Simo M Carpio C Iacoboni G Balaguer-Montero M Navarro V . PET-based radiomics signature can predict durable responses to CAR T-cell therapy in patients with large B-cell lymphoma. EJHaem. (2023) 4:1081–8. doi: 10.1002/jha2.757 PMC10660117 38024636

[34] Zhou Y Zhang B Han J Dai N Jia T Huang H . Development of a radiomic-clinical nomogram for prediction of survival in patients with diffuse large B-cell lymphoma treated with chimeric antigen receptor T cells. J Cancer Res Clin Oncol. (2023) 149:11549–60. doi: 10.1007/s00432-023-05038-w PMC11797235 37395846

[35] Gui J Li M Xu J Zhang X Mei H Lan X . [(18)F]FDG PET/CT for prognosis and toxicity prediction of diffuse large B-cell lymphoma patients with chimeric antigen receptor T-cell therapy. Eur J Nucl Med Mol Imaging. (2024). doi: 10.1007/s00259-024-06667-0 38467921

[36] Locke FL Oluwole OO Kuruvilla J Thieblemont C Morschhauser F Salles GA . Axicabtagene ciloleucel versus standard of care in second-line large B-cell lymphoma: outcomes by metabolic tumor volume. Blood. (2024). doi: 10.1182/blood.2023021620 PMC11208295 38557775

[37] Marchal E Palard-Novello X Lhomme F Meyer ME Manson G Devillers A . Baseline [(18)F]FDG PET features are associated with survival and toxicity in patients treated with CAR T cells for large B cell lymphoma. Eur J Nucl Med Mol Imaging. (2024) 51:481–9. doi: 10.1007/s00259-023-06427-6 37721580

[38] Rojek AE Kline JP Feinberg N Appelbaum DE Pu Y Derman BA . Optimization of metabolic tumor volume as a prognostic marker in CAR T-cell therapy for aggressive large B-cell NHL. Clin Lymphoma Myeloma Leuk. (2024) 24:83–93. doi: 10.1016/j.clml.2023.09.005 37827881

[39] Voltin CA Paccagnella A Winkelmann M Heger JM Casadei B Beckmann L . Multicenter development of a PET-based risk assessment tool for product-specific outcome prediction in large B-cell lymphoma patients undergoing CAR T-cell therapy. Eur J Nucl Med Mol Imaging. (2024) 51:1361–70. doi: 10.1007/s00259-023-06554-0 PMC10957657 38114616

[40] Meignan M Cottereau AS Specht L Mikhaeel NG . Total tumor burden in lymphoma - an evolving strong prognostic parameter. Br J Radiol. (2021) 94:20210448. doi: 10.1259/bjr.20210448 34379496 PMC8553180

[41] Cheson BD . PET/CT in lymphoma: current overview and future directions. Semin Nucl Med. (2018) 48:76–81. doi: 10.1053/j.semnuclmed.2017.09.007 29195620

[42] Chauvie S Ceriani L Zucca E . Radiomics in Malignant lymphomas. In: Lymphoma [Internet]. (Brisbane (AU): Exon Publications) (2021). 35226435

[43] Breen WG Young JR Hathcock MA Kowalchuk RO Thorpe MP Bansal R . Metabolic PET/CT analysis of aggressive Non-Hodgkin lymphoma prior to Axicabtagene Ciloleucel CAR-T infusion: predictors of progressive disease, survival, and toxicity. Blood Cancer J. (2023) 13:127. doi: 10.1038/s41408-023-00895-7 37591834 PMC10435575

[44] Ababneh HS Ng AK Abramson JS Soumerai JD Takvorian RW Frigault MJ . Metabolic parameters predict survival and toxicity in chimeric antigen receptor T-cell therapy-treated relapsed/refractory large B-cell lymphoma. Hematol Oncol. (2024) 42:e3231. doi: 10.1002/hon.3231 37795759

[45] Mehta PH Fiorenza S Koldej RM Jaworowski A Ritchie DS Quinn KM . T cell fitness and autologous CAR T cell therapy in haematologic Malignancy. Front Immunol. (2021) 12:780442. doi: 10.3389/fimmu.2021.780442 34899742 PMC8658247

[46] Noll JH Levine BL June CH Fraietta JA . Beyond youth: Understanding CAR T cell fitness in the context of immunological aging. Semin Immunol. (2023) 70:101840. doi: 10.1016/j.smim.2023.101840 37729825

[47] Kampouri E Little JS Rejeski K Manuel O Hammond SP Hill JA . Infections after chimeric antigen receptor (CAR)-T-cell therapy for hematologic Malignancies. Transpl Infect Dis. (2023) 25 Suppl 1:e14157. doi: 10.1111/tid.14157 37787373

[48] El-Galaly TC Villa D Cheah CY Gormsen LC . Pre-treatment total metabolic tumour volumes in lymphoma: Does quantity matter? Br J Haematol. (2022) 197:139–55. 10.1111/bjh.18016 35037240

[49] Kostakoglu L Chauvie S . Metabolic tumor volume metrics in lymphoma. Semin Nucl Med. (2018) 48:50–66. doi: 10.1053/j.semnuclmed.2017.09.005 29195618

