# Supplementary material for: Metabolic tumor volume and the survival of patients with Non-Hodgkin lymphoma treated with chimeric antigen receptor T cell therapy: a meta-analysis
Source: Front Immunol. 2024 Aug 29;15:1433012. doi: 10.3389/fimmu.2024.1433012 (PMC11390410; doi:10.3389/fimmu.2024.1433012)
Supplement: Supplementary file 1 [file Table1.docx]

**PubMed**

("Chimeric Antigen Receptor"[All Fields] OR "Chimeric Antigen Receptors"[All Fields] OR "chimeric t cell receptors"[All Fields] OR "chimeric t cell receptors"[All Fields] OR "Chimeric Antigen Receptor T Cell"[All Fields] OR "CAR-T"[All Fields] OR "artificial t cell receptors"[All Fields] OR "artificial t cell receptors"[All Fields] OR "chimeric immunoreceptors"[All Fields] OR "Axicabtagene ciloleucel"[All Fields] OR "Axi-cel"[All Fields] OR "KTE-C19"[All Fields] OR "KTEC19"[All Fields] OR "CTL-019"[All Fields] OR "CTL019"[All Fields] OR "Yescarta"[All Fields] OR "Lisocabtagene"[All Fields] OR "maraleucel"[All Fields] OR "Liso-cel"[All Fields] OR "JCAR017"[All Fields] OR "Breyanzi"[All Fields] OR "Brexucabtagene"[All Fields] OR "autoleucel"[All Fields] OR "Brexu-cel"[All Fields] OR "KTE-X19"[All Fields] OR "KTEX19"[All Fields] OR "Tecartus"[All Fields] OR "Tisagenlecleucel"[All Fields] OR "Tisa-cel"[All Fields] OR "Kymriah"[All Fields] OR "CART19"[All Fields] OR "Axicabtagene"[All Fields] OR "ciloleucel"[All Fields] OR "Idecabtagene"[All Fields] OR "vicleucel"[All Fields] OR "autoleucel"[All Fields]) AND ("lymphoma"[All Fields] OR "non-hodgkin lymphoma"[All Fields]) AND ("18F-FDG PET/CT"[All Fields] OR "positron emission tomography"[All Fields] OR "positron emission tomography-computed tomography"[All Fields] OR "pet ct"[All Fields] OR "PET"[All Fields] OR "pet ct"[All Fields] OR "pet ct"[All Fields] OR "fluorodeoxyglucose"[All Fields] OR "metabolic tumor volume"[All Fields] OR "MTV"[All Fields]) AND ("survival"[All Fields] OR "overall survival"[All Fields] OR ("mortality"[MeSH Subheading] OR "mortality"[All Fields] OR "survival"[All Fields] OR "survival"[MeSH Terms] OR "survivability"[All Fields] OR "survivable"[All Fields] OR "survivals"[All Fields] OR "survive"[All Fields] OR "survived"[All Fields] OR "survives"[All Fields] OR "surviving"[All Fields]) OR "OS"[All Fields] OR "PFS"[All Fields] OR "death"[All Fields] OR "mortality"[All Fields] OR "progression"[All Fields] OR "prognosis"[All Fields] OR "cohort"[All Fields] OR "longitudinal"[All Fields] OR "prospective"[All Fields] OR "retrospective"[All Fields] OR "followed"[All Fields] OR "follow-up"[All Fields])

**Embase**

('chimeric antigen receptor' OR 'chimeric antigen receptors' OR 'chimeric t cell receptors' OR 'chimeric t-cell receptors' OR 'chimeric antigen receptor t cell' OR 'car-t' OR 'artificial t cell receptors' OR 'artificial t-cell receptors' OR 'chimeric immunoreceptors' OR 'axicabtagene ciloleucel' OR 'axi-cel' OR 'kte-c19' OR 'ktec19' OR 'ctl-019' OR 'ctl019' OR 'yescarta' OR 'lisocabtagene' OR 'maraleucel' OR 'liso-cel' OR 'jcar-017' OR 'jcar017' OR 'breyanzi' OR 'brexucabtagene' OR 'brexu-cel' OR 'kte-x19' OR 'ktex19' OR 'tecartus' OR 'tisagenlecleucel' OR 'tisa-cel' OR 'kymriah' OR 'art-19' OR 'cart19' OR 'axicabtagene' OR 'ciloleucel' OR 'idecabtagene' OR 'vicleucel' OR 'ciltacabtegene' OR 'autoleucel') AND ('lymphoma' OR 'non-hodgkin lymphoma') AND ('18f-fdg pet/ct' OR 'positron emission tomography' OR 'positron emission tomography-computed tomography' OR 'pet-ct' OR 'pet' OR 'pet ct' OR 'pet/ct' OR 'fluorodeoxyglucose' OR 'metabolic tumor volume' OR 'mtv') AND ('survival' OR 'overall survival' OR 'progression-fress survival' OR 'os' OR 'pfs' OR 'death' OR 'mortality' OR 'progression' OR 'prognosis' OR 'cohort' OR 'longitudinal' OR 'prospective' OR 'retrospective' OR 'followed' OR 'follow-up') AND [abstracts]/lim AND [clinical study]/lim AND [humans]/lim AND [embase]/lim

**Web of Science**

("Chimeric Antigen Receptor" OR "Chimeric Antigen Receptors" OR "Chimeric T Cell Receptors" OR "Chimeric T-Cell Receptors" OR "Chimeric Antigen Receptor T Cell" OR "CAR-T" OR "Artificial T Cell Receptors" OR "Artificial T-Cell Receptors" OR "chimeric immunoreceptors" OR "Axicabtagene ciloleucel" OR "Axi-cel" OR "KTE-C19" OR "ktx19" OR "CTL-019" OR "CTL019" OR "yescartatm" OR "Lisocabtagene" OR "maraleucel" OR "Liso-cel" OR "JCAR-017" OR "jcar015" OR "brelandi" OR "Brexucabtagene" OR "autoleucel" OR "Brexu-cel" OR "KTE-X19" OR "ktx19" OR "telarius" OR "Tisagenlecleucel" OR "Tisa-cel" OR "Kymriah" OR "ART-19" OR "CART19" OR "Axicabtagene" OR "ciloleucel" OR "Idecabtagene" OR "vicleucel" OR "ciltacabtagene" OR "autoleucel") AND ("lymphoma" OR "non-Hodgkin lymphoma") AND ("18F-FDG PET/CT" OR "positron emission tomography" OR "positron emission tomography-computed tomography" OR "PET-CT" OR "PET" OR "PET CT" OR "PET/CT" OR "fluorodeoxyglucose" OR "metabolic tumor volume" OR "MTV") AND ("survival" OR "overall survival" OR "progression-fress survival" OR "OS" OR "PFS" OR "death" OR "mortality" OR "progression" OR "prognosis" OR "cohort" OR "longitudinal" OR "prospective" OR "retrospective" OR "followed" OR "follow-up") (Topic)
